# TSPDB: a curated resource of tailspike proteins with potential applications in phage research

**DOI:** 10.3389/fdata.2024.1437580

**Published:** 2024-11-27

**Authors:** Opeyemi U. Lawal, Lawrence Goodridge

**Affiliations:** Canadian Research Institute for Food Safety (CRIFS), Department of Food Science, University of Guelph, Guelph, ON, Canada

**Keywords:** phage, tailspike proteins, genomics, big data, data mining

## Background

Bacteriophages (phages) are viruses that infect and replicate within host bacteria and archaea (Chatterjee and Duerkop, 2018; Dion et al., 2020). Phages are the most abundant entities in the biosphere (Dion et al., 2020) and are distributed across different biomes populated by bacterial and archaeal hosts, including the gastrointestinal tract of humans and animals, and oceanic beds (Chevallereau et al., 2022; Clokie et al., 2011). They play a vital role in the rapid evolution and adaptation of their hosts in various environments (Dion et al., 2020).

Phages exhibit high genomic, morphological, and structural diversity, composed of DNA or RNA that can be single-stranded or double-stranded and packaged into a capsid (Dion et al., 2020; Fokine and Rossmann, 2014). The structural form of the capsid was a major feature used in the taxonomic classification of phages until the advent of whole-genome sequencing, which has now become the gold standard for this classification (Dion et al., 2020; Fokine and Rossmann, 2014; Turner et al., 2023). Phages are broadly classified as tailed or non-tailed, with double-stranded DNA tailed phages constituting about 96% of all known phages (Dion et al., 2020). Phages possess a diverse array of tail structures essential for host recognition, attachment, and penetration, making them important targets in phage therapy research (Fokine and Rossmann, 2014; Gil et al., 2023). Phage infection of its host begins with the recognition of a receptor on the bacterial cell surface for attachment (Dowah and Clokie, 2018; Latka et al., 2017). To penetrate the host cell, phages must overcome various complex barriers on the bacterial cell wall, such as the outer membrane of Gram-negative bacteria and the lipoteichoic acids of Gram-positive bacteria (Chen et al., 2014; Latka et al., 2017). Phages encode virion-associated carbohydrate-degrading enzymes called depolymerases, which are distinct from the endolysins produced by phages during the lysis stage (Knecht et al., 2020; Yan et al., 2014). These depolymerases, encoded by tailspike protein (TSP) genes, recognize, bind, and degrade cell-surface associated polysaccharides, unmasking phage receptors and making them accessible for bacterial infection (Gil et al., 2023; Greenfield et al., 2019; Latka et al., 2017).

Tailspike proteins are integral components of phage tail structures, and their activities as polysaccharide depolymerases are related to host specificity and infectivity (Greenfield et al., 2019). A hallmark of TSPs is their host specificity, high thermostability, resistance to protease treatment, and stability in the presence of high concentrations of urea and sodium dodecyl sulfate (Chen et al., 2014). Phage TSPs possess carbohydrate depolymerase activity and recognize capsule, and lipopolysaccharides (LPS) where they cleave components of the LPS to position the phage toward a secondary membrane receptor during infection (Knecht et al., 2020). TSPs have been observed to decrease bacterial viability, leading to antimicrobial applications. For example, Ayariga et al. (2021) demonstrated that the ε34 phage tailspike protein has enzymatic property as a LPS hydrolase and synergizes with Vero Cell culture supernatant in killing *Salmonella* Newington. The ε34 TSP also showed bactericidal efficacy against different *Salmonella* serovars in various matrices (Ibrahim et al., 2023). Miletic and colleagues (Miletic et al., 2016) expressed the receptor binding domain of the Phage P22 Gp9 tailspike protein in plant tissue (*Nicotiana benthamiana*), and demonstrated that, upon oral administration of lyophilized leaves expressing Gp9 TSP to newly hatched chickens, *Salmonella* concentrations were reduced on average by approximately 0.75 log relative to controls. Others have shown that TSPs can be used to control the growth of plant pathogens. For example, expression of the *Erwinia* spp. phage TSP DpoEa1h in transgenic apple and pear plants significantly reduced fire blight (*Erwinia amylovora*) susceptibility (Malnoy et al., 2005; Roach and Donovan, 2015), likely due to removal of the main virulence factor amylovoran and exposing the *E. amylovora* cells to host plant defenses (Kim et al., 2004). Finally, phage LKA1 TSP exhibits disruptive activity against biofilms while also reducing virulence in *Pseudomonas* in an infection model (Olszak et al., 2017). Collectively, these studies demonstrate the utility of TSPs as novel antimicrobials to control the growth of food and plant-borne pathogens in foods.

Despite the known antimicrobial applications of TSPs, only a few have been fully characterized to date. This could be partly due to the laborious nature of detection techniques, which include plaque assays followed by examination under a transmission electron microscope (TEM) to identify “bulb-like” baseplate structures at the base of phage tails indicative of TSPs (Bhandare et al., 2024; Knecht et al., 2020). The decreasing costs of sequencing and the availability of improved bioinformatics tools have facilitated the construction of large-scale genome and metagenome datasets (Emond-Rheault et al., 2017; Wattam et al., 2014). High-throughput *in silico* detection of TSP-encoding genes in genomic data would not only provide further details regarding the diversity of TSPs in virulent phages but could also be used to identify TSPs in prophages. In this report, we present a high-level curated resource called TSP database (TSPDB) for the rapid detection of tailspike proteins in multiomics sequence data. This TSPDB will be an indispensable resource for researchers in phage biology, drug discovery, and antimicrobial resistance domains to further contribute to the understanding of the structure and function of these proteins to harness their potential for diverse applications, such as the development of phage therapy for bacterial infections or phage-based biocontrol of foodborne pathogens, and drug discovery (Brives and Pourraz, 2020; Roach and Donovan, 2015).

## Data and methodology

### Data mining and quality check

**Figure 1:**
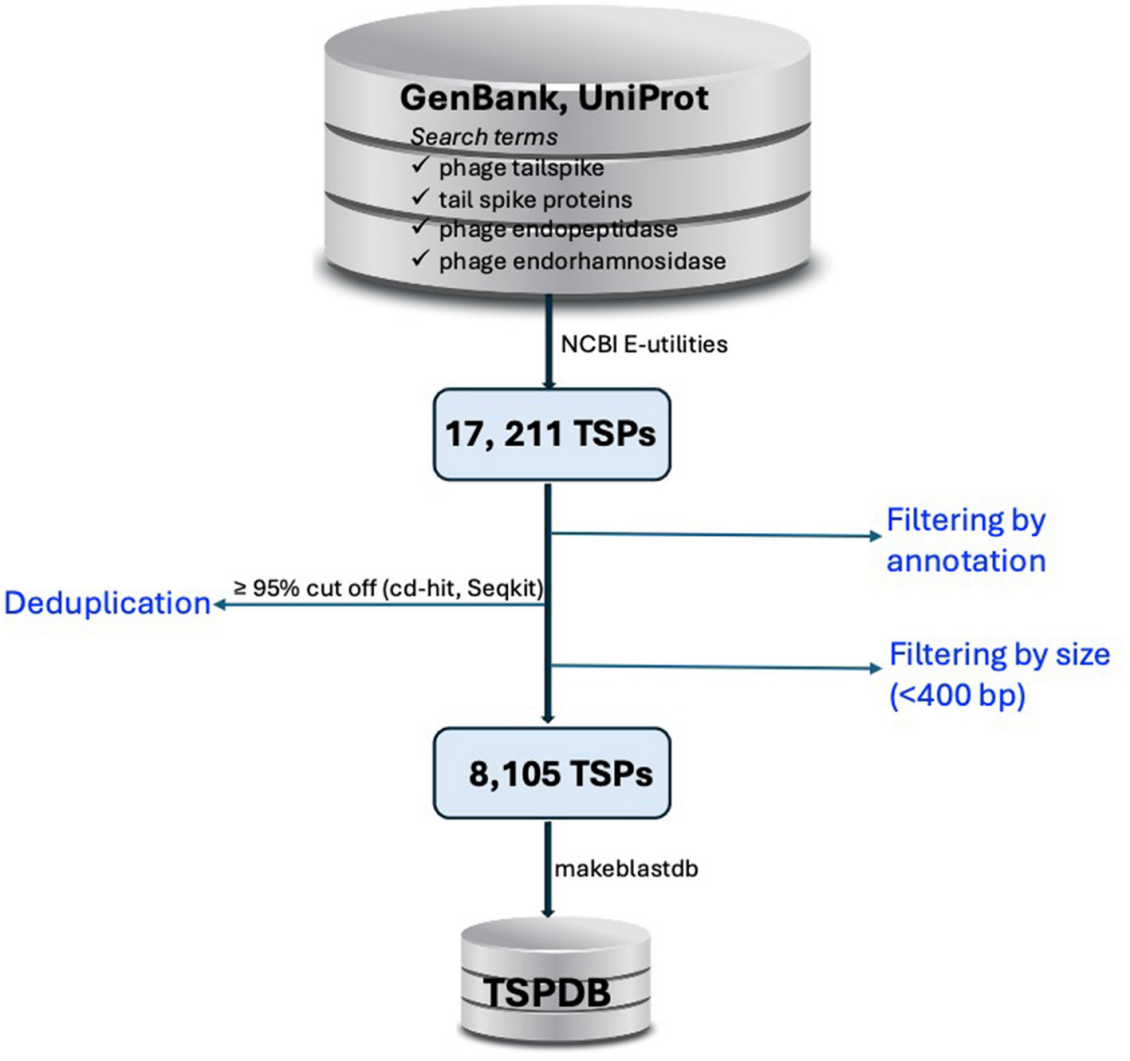
Workflow for the construction of the tailspike protein database (TSPDB). TSP sequences were retrieved from GenBank and UniProt using specific search terms, yielding 17,211 sequences. After filtering by annotation and excluding sequences <400 bp and >10,000 bp, deduplication at ≥95% similarity reduced the dataset to 8,105 unique sequences, which were then compiled into the TSPDB for efficient access.

The DDBJ/ENA/GenBank and UniProt databases (Sayers et al., 2022; The UniProt Consortium et al., 2023) were queried for TSPs using search terms commonly associated with tailspike proteins, such as “phage tailspike,” “tail spike proteins,” “phage endopeptidase,” and “phage endorhamnosidase.” (Figure 1). Hits were systematically filtered based on annotation criterion to exclude duplicate results. Nucleotide sequences of TSPs were retrieved from public databases using accession numbers obtained from the database query via NCBI Entrez Programming Utilities (E-utilities) (National Center for Biotechnology Information, 2023).

### Dataset curation

**Figure 2:**
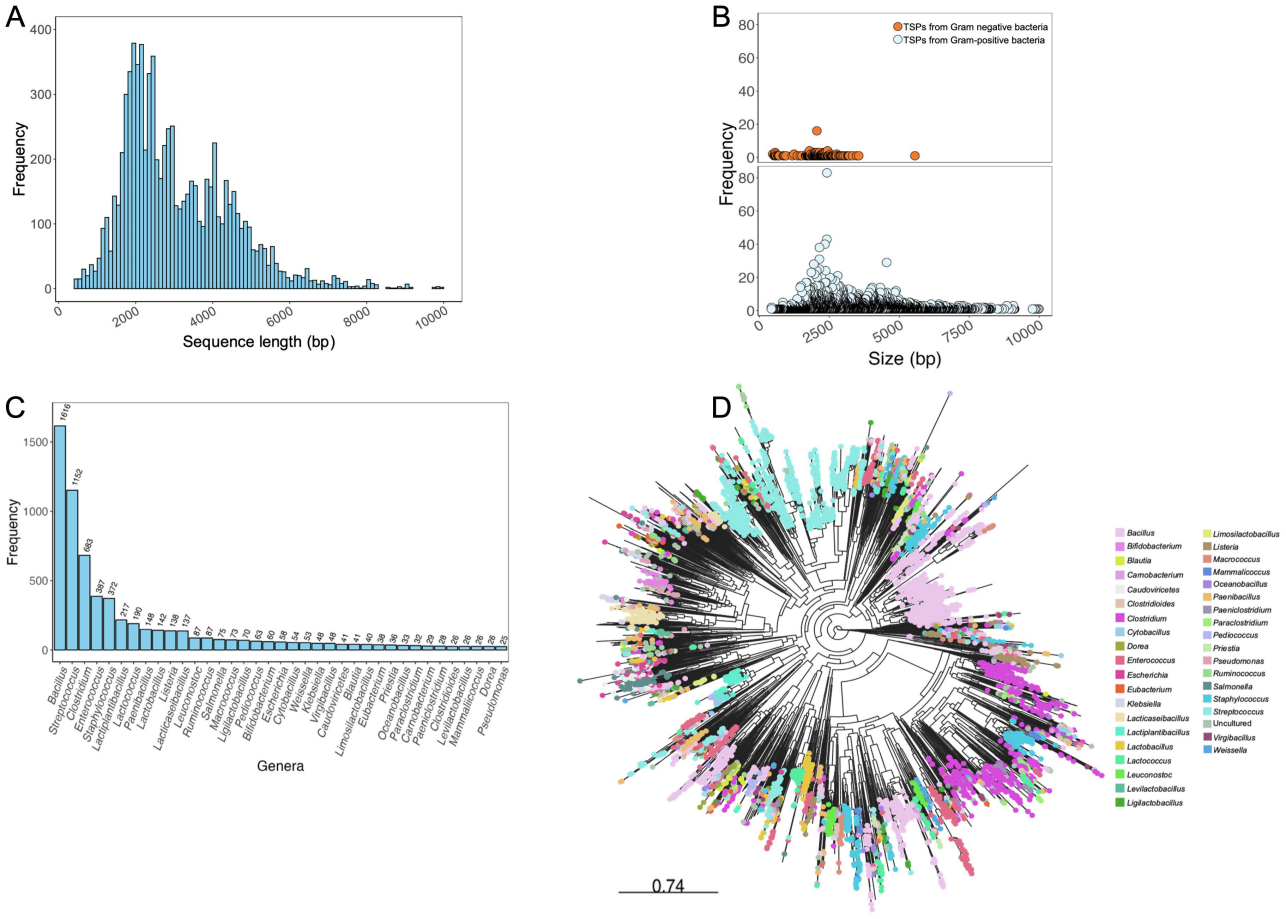
Analysis of phage tail spike proteins in the TSPDB. **(A)** Sequence length distribution of genes encoding phage TSPs contained in the TSPDB. TSP size in the database ranged from 405–9,990 bp. **(B)** Differential frequency distribution of TSP gene sizes in Gram-negative (orange circles) and Gram-positive (blue circles) bacteria in the TSPDB. **(C)** Frequency across the top 37 genera of host phages carrying TSPs in the TSPDB. **(D)** Phylogenetic diversity of the 8,105 TSPs in the TSPDB. Each node represents a unique TSP contained in the TSPDB, with nodes of similar color belonging to the same genera. The top 37 genera are displayed in color. An interactive version of this figure is accessible through the following link - https://microreact.org/project/7Kv61nb6aRapgGgHpxsNGL-tspdb-v20. To assess the normality of the distribution of TSP frequencies across bacterial genera, we performed a Shapiro-Wilk test. In this analysis, the Shapiro-Wilk test yielded a statistic of 0.487 and a *p*-value < 0.0001, confirming a significant departure from normality. This result supports the observation of a skewed distribution, where a small number of genera (e.g., *Bacillus* and *Streptococcus*) exhibit notably high TSP counts compared to others.

From this exercise, 17,211 sequences were obtained from the queried public databases. Duplicated sequences were removed using thresholds of ≥95% sequence coverage and nucleotide similarity with cd-hit (Li and Godzik, 2006) and Seqkit (Shen et al., 2016), resulting in 9,129 unique TSP sequences. To assess the sequence length distribution and perform quality checks on unique TSP sequences, Gaussian distribution analysis was conducted. Sequences shorter than 400 bp, which could represent partial region or incomplete sequences that may lack critical functional domains required for accurate annotation and functional prediction, were excluded from the dataset. By excluding these shorter sequences, we reduce the possibility of including fragments that could introduce noise or inaccuracies into the database. This threshold helps ensure that the TSPDB contains more reliable and complete sequences for functional analysis and annotation. This filtering process resulted in a total of 8,105 unique TSP sequences (Figure 1). TSP sequences with a length of ≤ 10,000 bp were retained to include those originating from Gram-positive bacteria such as *Clostridium* and *Streptococcus*, among others. Overall size range of TSPs retrieved from the public databases is 405 to 9,990 bp (Figure 2A). Further analysis of TSP genes in the TSPDB reveals a significant difference (*p* < 0.001) in the sizes of TSPs between Gram-negative and Gram-positive bacteria. Specifically, the average size of TSPs for Gram-negative bacteria is 2,070 bp, while the average size for Gram-positive bacteria is substantially larger, at 3,255 bp (Figure 2B). The TSPDB contains TSPs from more than 400 bacterial genera. Among these, the top 13 genera represented were Gram-positive bacteria, with TSPs from *Bacillus* (*n* = 1,616) being the most common, followed by *Streptococcus* (*n* = 1,152), *Clostridium* (*n* = 683), *Enterococcus* (*n* = 387), and *Staphylococcus* (*n* = 372). Additionally, TSPs from Gram-negative bacterial genera, *Salmonella* (*n* = 80), *Escherichia* (*n* = 58), *Klebsiella* (*n* = 52), and *Pseudomonas* (*n* = 25) were among the top 38 TSPs in the database (Figure 2C). To assess the normality of the distribution of TSP frequencies across bacterial genera, we performed a Shapiro-Wilk test. This test yielded a statistic of 0.487 and a *p*-value < 0.0001, confirming a significant departure from normality. This result supports the observation of a skewed distribution, where Gram positive bacteria host genera (e.g., *Bacillus* and *Streptococcus*) exhibit notably high TSP counts compared to others.

### Diversity of TSPs

To assess the diversity of the 8,105 unduplicated TSP sequences and their suitability for database creation, we employed a phylogeny-based approach. The TSP sequences were aligned using MAFFT v7.453 (Katoh, 2002), and a maximum likelihood tree was constructed with FastTree v2.1.11 (Price et al., 2010) using the generalized time reversible mode and 1,000 bootstrap replicates for node support. The resulting phylogenetic tree was visualized using the web-based Microreact visualization tool (Argimón et al., 2016) (Figure 2D). The phylogeny revealed the high diversity of TSPs in the TSPDB, further supporting the uniqueness of individual TSPs. TSPs from the same species often belonged to different clusters. For example, TSPs from *Bacillus* and *Listeria* were distributed across multiple clusters in the phylogeny. While the majority of TSPs from *Salmonella* belonged to the same cluster, there were also a few instances of TSPs from this host genus in separate clusters (Figure 2D).

### TSPDB construction

The deduplicated TSP nucleotide sequences were utilized to construct the TSP database using makeblastdb (Camacho et al., 2009). This database is compatible for use with ABRicate (https://github.com/tseemann/abricate) and other bioinformatics tools equipped with embedded BLAST algorithms, such as BLAST suites and SRST2 (Inouye et al., 2014), among others.

### TSPDB application

The TSPDB was recently utilized in a study by Bhandare et al. (2024), where the database was implemented within an ABRicate container. The database index files suitable for use with blast was generated using makeblast_db option in ABRicate. The step-by-step guide on how to incorporate TSPDB into ABRicate for rapid screening of large genomic dataset is detailed on the ABRicate Github page (https://github.com/tseemann/abricate). The presence of TSPs in a collection of phage genomes were determined using stringent parameters (≥90% identity and ≥70% coverage). TSPDB provides valuable applications across various fields, particularly in phage therapy, biocontrol, and functional genomics and would contribute to advancing the application of TSPs in biocontrol strategies in agriculture and food safety. Overall, the TSPDB contains a vast dataset of diverse TSPs found in phages, and the integration of this database into phage detection tools will enhance the functional annotation of these genes in large genomic and metagenomic datasets. Lastly, the TSPDB described here will undergo regular updates and expansion to include new TSPs as they become available in public databases ensuring that the database remains comprehensive.

### Limitations

It is acknowledged that mis-annotation of some TSPs as hypothetical proteins or tail fibers in public databases may have resulted in the omission of certain TSP genes in this study. However, the TSPDB will be continually updated to incorporate additional TSP genes.

### Dataset description

The TSPDB is freely accessible on GitHub at the following link: https://github.com/yemilawal/Tailspike-proteins or by searching for the title “TSPDB: A curated resource of tailspike proteins with potential applications in phage research” on GitHub. Additionally, accession numbers of genes encoding phage tailspike proteins in TSPDB are available on the GitHub page. A backup version is also available for download on Figshare at https://doi.org/10.6084/m9.figshare.25526323.

## Data Availability

The original contributions presented in the study are included in the article/supplementary material, further inquiries can be directed to the corresponding authors.
